# Study of the effect of keap1 on oxidative stress in human umbilical cord mesenchymal stem cells

**DOI:** 10.1007/s11033-023-08997-y

**Published:** 2024-01-03

**Authors:** Hongrong Deng, Yunxia Chen, Huiwen Liu, Li Wang, Hao Xu, Bin Tan, Qin Yi, Rui Wang, Bolin He, Jie Tian, Jing Zhu

**Affiliations:** 1 Department of Pediatric Research Institute, National Clinical Research Center for Child Health and Disorders, Children’s Hospital of Chongqing Medical University, Ministry of Education Key Laboratory of Child Development and Disorders,Chongqing Key Laboratory of PediatricsChongqing Key Laboratory of Pediatrics, Chongqing, China; 2 Department of Clinical Laboratory, Children’s Hospital of Chongqing Medical University, Chongqing, China; 3 Department of Blood Transfusion, Children’s Hospital of Chongqing Medical University, Chongqing, China; 4 Department of Cardiovascular Internal Medicine, Children’s Hospital of Chongqing Medical University, Chongqing, China

**Keywords:** keap1, Oxidative stress, HucMSCs, IKKβ

## Abstract

**Background:**

HucMSCs had shown promising efficacy in treating childhood diseases, but oxidative stress induced by the poor microenvironment at the site of damage resulted in low cell survival after transplantation, thus preventing the cells from maximizing therapeutic efficacy. Therefore, this study aimed to investigate the role and mechanism of keap1 in oxidative stress injury of human umbilical cord mesenchymal stem cells (hucMSCs), and to provide theoretical support for improving the efficacy of stem cell therapy.

**Methods:**

The hucMSCs were treated with hypoxic low-sugar-free serum (GSDH) to mimic the damaged site microenvironment after implantation. Adenoviral overexpression of keap1 gene of hucMSCs was performed in vitro, and cell proliferation ability was detected by CCK8 assay, crystal violet staining assay, and cell cycle assay. Cellular redox level was assessed by Amplex Red, MDA, and GSH/GSSG kit. Mitochondrial morphology was evaluated by mitotracker Red staining. ATP production was estimated by ATP detection kit. The mRNA and protein expression levels were tested by western blotting and RT-qPCR.

**Results:**

GSDH treatment substantially upregulated keap1 expression. Subsequently, we found that overexpression of keap1 notably inhibited cell proliferation and caused cells to stagnate in G1 phase. At the same time, overexpression of keap1 induced the production of large amounts of H_2_O_2_ and the accumulation of MDA, but suppressed the GSH/GSSG ratio and the expression of antioxidant proteins NQO1 and SOD1, which caused oxidative stress damage. Overexpression of keap1 induced cells to produce a large number of dysfunctional mitochondria resulting in reduced ATP production. Moreover, Overexpression of keap1 significantly decreased the IKKβ protein level, while upregulating IkB mRNA levels and downregulating P50 mRNA levels.

**Conclusions:**

Overexpression of keap1 may induce oxidative stress injury in hucMSCs by down-regulating IKKβ expression and inhibiting NF-κB pathway activation. This implies the importance of keap1 in hucMSCs and it may be a potential gene for genetic modification of hucMSCs.

## Introduction

Human umbilical cord mesenchymal stem cells (hucMSCs), with their properties of self-renewal, multidirectional differentiation and immunomodulation, have received widespread attention in modern medicine. Non-invasive access, higher proliferative capacity, lower immunogenicity, and the absence of ethical issues make them an ideal source for stem cell transplantation therapy [1, 2]. Many preclinical studies and clinical trials have been carried out with good success in HucMSCs for the treatment of a wide range of pediatric diseases [3, 4]. Interestingly, one study showed that MSCs were more effective in pediatric patients than in adult patients [5]. Currently, commercialized MSCs have been approved by the FDA in Japan for pediatric steroid-refractory GVHD [6]. However, although hucMSCs have achieved good efficacy in clinical trials, we still face great challenges. Differences in oxygen concentration and nutritional conditions between in vivo and in vitro may subject hucMSCs to high oxidative stress after transplantation in vivo, reducing colonization and survival rates and preventing effective treatment of damaged sites [35, 7, 8]. Therefore, it is particularly important to explore the molecular mechanisms that cause oxidative stress in hucMSCs, find intervening targets, and genetically modify MSCs to improve therapeutic efficacy in pediatric diseases.

Oxidative stress is a state in which the oxidative and antioxidant systems are in an imbalance, which can interfere with a variety of signaling pathways and thus affect biological processes [9]. Therefore, Maintaining the homeostasis of the redox system is therefore essential for the normal biological functioning of cells. Kelch-like ECH-associated protein 1 (keap1) is an oxidative stress sensor. Studies have shown that when cells undergo oxidative stress, keap1 cysteine residues are oxidized, which upregulates the transcript levels of antioxidant proteins [10]. Z Fan et al. [11] showed that overexpression of keap1 inhibited cell proliferation and caused cell death. It was shown that inhibition of keap1 expression in a hepatic ischemia-reperfusion injury model protects against ischemia-reperfusion-induced oxidative stress injury by upregulating the expression of antioxidant proteins [12]. Meanwhile, it has been shown that inhibition of keap1 promotes BMSC proliferation ginsenosides [13]. However, little is known about the relationship between keap1 and causing oxidative stress in hucMSCs.

Keap1 is a ubiquitin ligase that degrades IKKβ, thereby inhibiting the activation of the NF-κB pathway [14]. The NF-κB pathway regulates redox homeostasis, and its activation requires IKKβ, which is a protein kinase that phosphorylates and ubiquitinates IκB (κB inhibitor that binds to NF-κB subunits and inhibits their entry into the nucleus), resulting in translocation of NF-κB subunits (P50, P65) into the nucleus and initiation of target protein transcription [15]. Chen et al. [16] found that knockdown of IKKβ resulted in the accumulation of reactive oxygen species(ROS), causing cellular oxidative stress injury. It has been shown that inhibiting the degradation of IKKβ by keap1 may improve cell survival and proliferation [14]. Therefore, in hucMSCs, keap1 may mediate oxidative stress injury by regulating IKKβ. The aim of this study was to preliminarily investigate the effect of keap1 on oxidative stress in hucMSCs, and to provide theoretical support for subsequent genetic engineering of hucMSCs to improve therapeutic efficacy.

## Materials and methods

### Culture and characterization of hucMSCs

DMEM/F12 (Gibco) growth medium containing 10% fetal bovine serum (Cell max) was added to hucMSCs and placed in a 37 °C, 5% CO_2_, humidified incubator. HucMSCs were obtained from Chongqing Stem Cell Therapy Engineering and Technology Research Center. Cells were passaged and cultured to the 3rd generation, and after the cells reached 40%–50% confluence, hucMSCs were infected with GFP-tagged adenovirus with or without overexpression of keap1 for 24 h, and then the fluid was changed to continue the culture for 12 h.

When the cells were in the exponential growth phase, trypsin (Beyotime) digestion and centrifugation were performed and incubated with surface markers (BD) for 1 h away from light. Then flow cytometry was performed on the machine and Flow Jo was used for quantitative analysis.

### Crystalline violet staining

Crystal violet staining solution (Beyotime) was applied after the cells had been fixed with 4% paraformaldehyde (Beyotime) for 30 min. The cells were then stained at room temperature for 2 h. Images were captured using an inverted microscope. Cell was decolorized by adding 95% ethanol, and the absorbance was measured using a microplate reader at 570 nm.

### Annexin V/7AAD measurement

The cell precipitates were resuspended by adding 500 µl Binding Buffer, 5 µl Annexin V-APC and 5 µl 7AAD (KeyGEN BioTECH), respectively, mixed with slight oscillation, and then incubated at room temperature away from light for 30 min, then tested on the machine. Quantitative analysis was performed by Flow Jo.

### Cell immunofluorescence

The cells were fixed with 4% paraformaldehyde (Beyotime) for 30 min, treated with 0.5% Triton (Beyotime) for 10 min, then closed with 0.5% BSA (Solarbio) for 30 min at room temperature. The closure solution was discarded, and the cells were then incubated with the primary antibody overnight at 4 °C. The cells were treated for one hour at room temperature with fluorescent secondary antibody dilution (Abbkine), shielded from light, and washed three times with PBS. PBS was blotted dry, and appropriate amount of anti-fluorescence quencher (Solarbio) was added to cover the bottom of the confocal dish. Place it under a confocal microscope, observe and take pictures. Images were analyzed using Image J.

### Cell cycle detection

1 × 10^6^ cells were fixed by 0.5 ml 70% cold ethanol at 4 °C overnight. Each sample requires 0.5 ml of assay working solution (KeyGEN BioTECH), according to 9 µl Rnase: 1 µl PI to prepare the appropriate amount of assay working solution, incubated at room temperature and protected from light for 45 min, and then measured on the machine, using Flow Jo for data analysis.

### CCK8 assays

3000 cells were inoculated in 24-well plates and treated with adenovirus infection for 36 h. The working solution was prepared by adding 100 µl of working solution to each well in the ratio of 90 µl of basal medium to 10 µl of CCK8 (GLPBIO), and the cells and the working solution reacted for 3 h in an incubator, and then the OD value was detected by a microplate reader at 450 nm.

### Amplex red

Cells were adjusted to a single cell suspension of 7.5 × 10^5^ cells/ml. 100 µl of working solution (Thermo Fisher SCIENTIFIC) was added to each well of a white 96-well plate and placed in the incubator to preheat for 10 min. After adding 20 µl of PBS or samples to each well, the plate was placed and assayed on a microplate reader, with the parameters set to an excitation wavelength of 530 nm and an emission wavelength of 590 nm.

### MDA detection

The required reagents (Beyotime) were prepared according to the steps in the instructions. After the cells were lysed by the lysate, the total cell protein was extracted, and the protein concentration was determined. 200 µl of MDA working solution was added into a 1.5 ml EP tube, then mixed with 100 µl of PBS, standards, or samples. It was heated at 100 °C for 15 min, cooled to room temperature in a water bath, and centrifuged at 10,000*g* for 10 min at room temperature. 200 µl of supernatant was added into a 96-well plate, and the absorbance was measured at 532 nm on the microplate reader. The MDA content was calculated according to the instructions.

### GSH/GSSG measurement

All the required solutions (Beyotime) were prepared strictly, according to the instructions of the kit. Cell precipitates were collected and resuspended in 20 µl of PBS, and the cells were gently blown to disperse. 60 µl of Protein Removal Reagent M solution was added and mixed well. The samples were frozen and thawed twice at 37 °C and in liquid nitrogen, allowed to stand on ice for 5 min and centrifuged at 10,000*g* at 4 °C for 10 min. The supernatant was collected and divided into two portions, one portion to be used for the detection of total glutathione and one portion to be used for the detection of the GSSG content according to the following procedure. Samples of each tube were mixed with 5 µl of diluted GSH Scavenging Aid, followed by 1 µl of GSH Scavenging Reagent Working Solution and mix and then reacted at room temperature for 1 h to completely remove GSH and obtain GSSG. Aspirate 10 µl of M solution, standard, or sample was aspirated into a 96-well plate, followed by 150 µl of Total Glutathione Assay Working Solution, incubated at room temperature for 5 min. 50 µl NADPH (0.5 mg/ml) was added, mixed and measured for the absorbance A1 at 412 nm immediately with a microplate reader, and the absorbance A2 again after 30 min. The GSH/GSSG was calculated according to the instructions.

### Western blots

Total cellular proteins were extracted using the Total Protein Extraction Kit (KeyGEN BioTECH), and their concentrations were subsequently determined. The protein up-sampling volume was calculated based on the protein concentration. After the protein samples were electrophoresed by SDS-PAGE gel electrophoresis, they were electrotransferred at 14 V for 8–9 h onto a PVDF membrane (Millipore), which was closed with 5% skimmed milk powder for 2 h. Subsequently, the membrane was subjected to primary antibody dilutions. The sample was incubated at room temperature for 1 h in a dilution of HRP-labeled secondary antibody, and then rinsed with TBST before being developed. The grayscale values were analyzed by Image Lab. Information on the antibodies used in the study is displayed in Table 1.

**Table 1:** Antibodies information

	Antibodies	Manufacturer	Catalog nubbers	Dilution
WB	Anti-keap1	Cell signaling technology	4678 S	1:1000
	Anti-IKKβ	NOVUS	NBP2-03319	1:500
	Anti-SOD1	HUABIO	ER1706-49	1:500
	Anti-NQO1	HUABIO	ET1702-50	1:500
	Anti-BAX	Proteintech	50599-2-Ig	1:1000
	Anti-cytochrome C	HUABIO	ET1610-60	1:500
	Anti-rabbit IgG	Proteintech	SA00001-2	1:5000
	Anti-mouse IgG	Proteintech	SA00001-1	1:5000
	Anti-β-actin	ZEN-BIOSCIENCE	200068-8F10	1:2000
IF	Anti-keap1	Cell signaling technology	4678 S	1:200
	Anti-IKKβ	NOVUS	NBP2-03319	1:200
	Cy3 Goat Anti-Rabbit IgG	Beyotime	A0516	1:500
	Alexa Fluor 647-Goat Anti-mouse IgG	Beyotime	A0473	1:500

### ATP measurement

Cells were lysed by 200 µl ATP lysis solution (Beyotime), centrifuged at 4 °C at 12,000*g* for 15 min, and then took the supernatant. The standard and ATP working solution were prepared according to the instructions. 100 µl of ATP working solution was added into each well of a 96-well plate, standing for 5 min,and then 20 µl of samples or standard was added, mixed, and then tested on a chemiluminescence instrument immediately.

### Mitochondria detection

Cells were incubated with 500 µl 20 nmol/l Mitotracker Red working solution (Beyotime) in the incubator for 30 min, washed with PBS for 3 times and then incubated with 500 µl Hoechst 33342 reagent at 37 °C for 15 min. The cells were washed with PBS and then observed and took pictures under the confocal microscope.

### RT-qPCR

Cellular RNA was extracted by the AG RNAex Pro Reagent (Accurate Biology) and then reverse transcribed into cDNA according to the instructions (ABclonal). Reverse transcription conditions were 37 °C for 2 min, 55 °C for 15 min, 85 °C for 5 min and 4 °C for hold. And qPCR reaction conditions were 95 °C for 3 min; entry cycles of 95 °C for 5 s and 60 °C for 30 s were performed for 40 cycles. The 2^−ΔΔCT^ method was used for analysis with β-actin as the internal reference control. Detailed primers are listed in Table 2.

**Table 2:** Primer information

Genes	Primer sequence forward (5′–3′)	Primer sequence reverse (3′–5′)
β-actin	CCTGGCACCCAGCACAAT	GGGCCGGACTCGTCATAC
keap1	AGGTATGAGCCAGAGCGGGATG	CGGCATAAAGGAGACGATTGAGGAC
P65	CTGCCGCCTGTCCTTTCTCATC	ATGTCCTCTTTCTGCACCTTGTCAC
P50	CACTGTAACTGCTGGACCCAAGG	CGCCTCTGTCATTCGTGCTTCC
IκB	ACTCCCGACACCAACCATACCC	GTCCTCCTCACTCTCCTCTTCTTCC

### Statistical analysis

GraphPad Prism 7.0 software was used to statistically analyze the data.The data were tested for normality, and data that met the normal distribution were compared between two groups using the two independent samples *t* test. Data that didn’t meet the normal distribution were compared between the two groups using the Mann–Whitney test. p < 0.05 indicated statistical significance.

## Results

### HucMSCs expressed positive markers

**Fig. 1:**
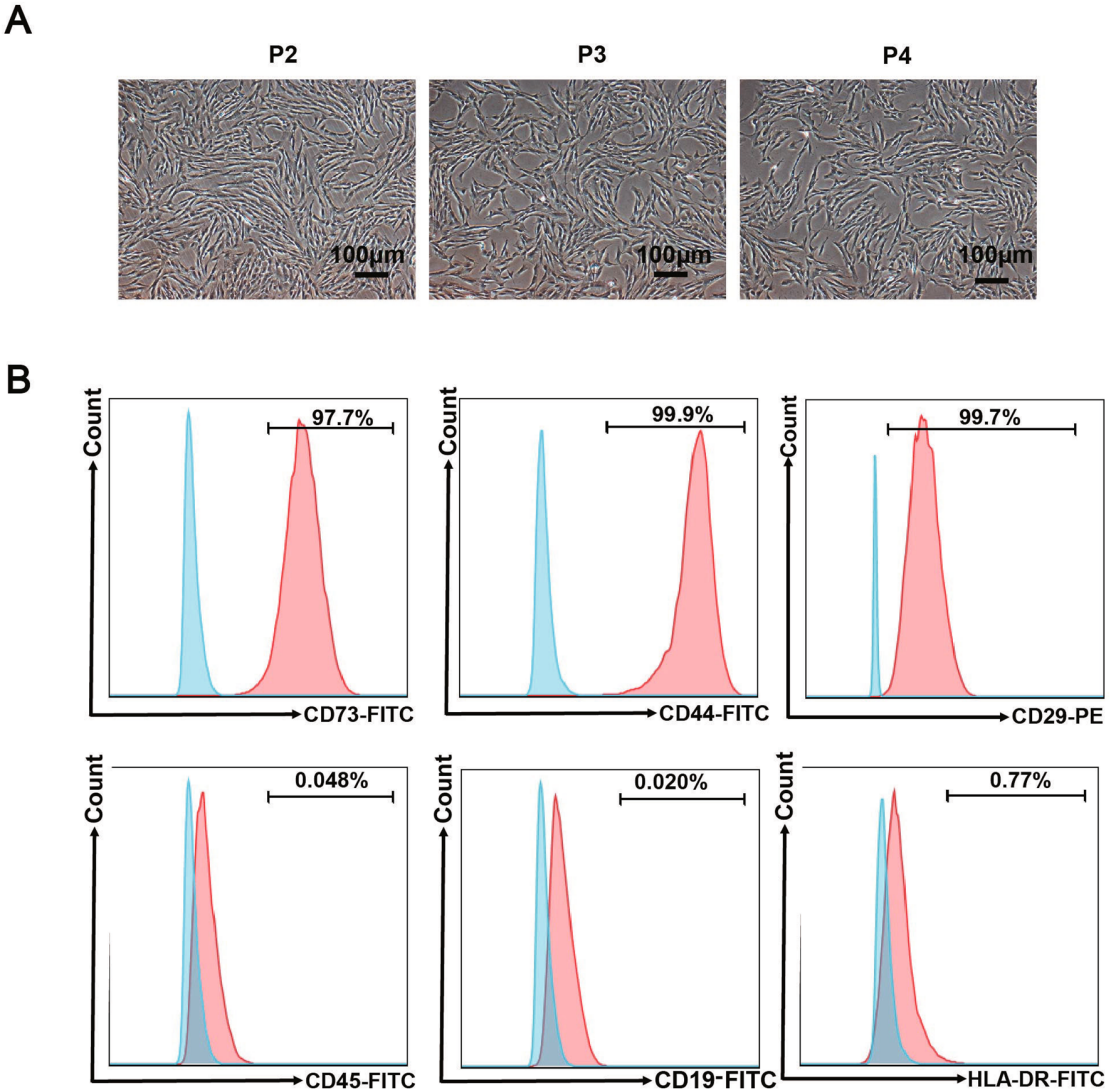
Identification of hucMSCs. **A** Morphological characterization of hucMSCs, scale bar: 100 μm. **B** Flow identification of hucMSCs, n = 3

Under the microscope, hucMSCs were observed to be pike-shaped and grew adherently to the wall (Fig. 1A). Flow results showed that hucMSCs expressed positive markers CD73, CD44, CD29 and did not express negative markers CD45, CD19, HLA-DR (Fig. 1B).

### GSDH treatment upregulated the expression of keap1

**Fig. 2:**
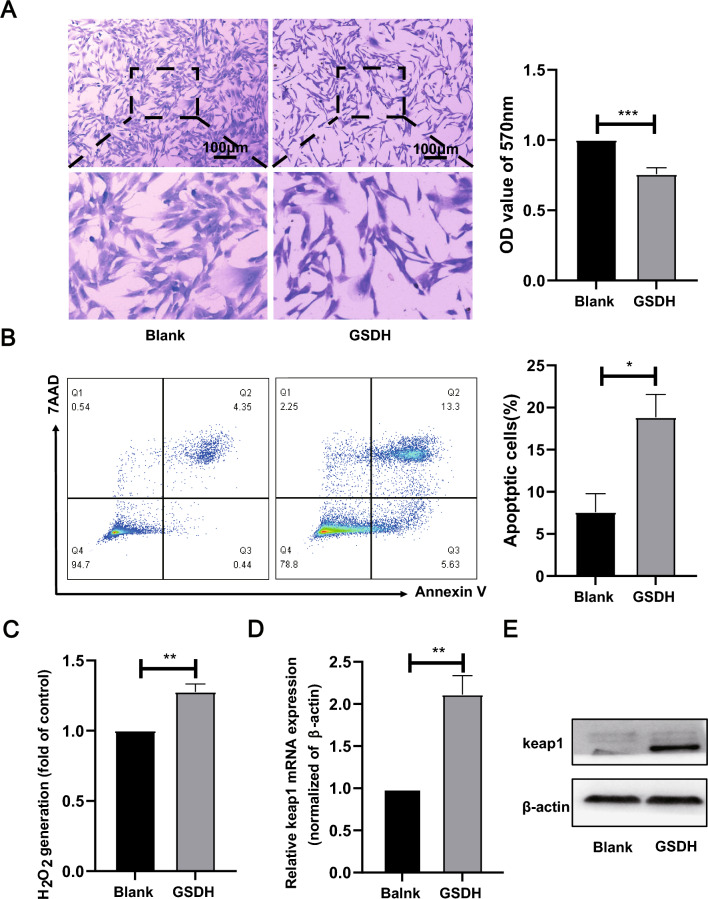
Construction of the GSDH model. **A** Crystalline violet staining was used to determine cell proliferation and quantified. p = 0.0008. Scale bar: 100 μm. **B** Annexin V/7AAD assay for cell apoptosis and quantified. p = 0.0323. **C** Amplex Red was detected for cellular H_2_O_2_ production. p = 0.0011. **D** RT-qPCR was performed to detect the level of keap1 mRNA. p = 0.0078. **E** WB was performed to detect the level of keap1 protein

Cells were treated with low oxygen (1% O_2_), low sugar (1.0 g/l), and serum-free medium to mimic the microenvironment of hucMSCs after transplantation into the damaged site. Compared with the Blank group, the GSDH group showed a remarkable decrease in total cells number (Fig. 2A), a notable increase in apoptotic cells (Fig. 2B,), and a significant increase in H_2_O_2_ production (Fig. 2C). Meanwhile, both mRNA and protein levels of keap1 were significantly higher in the GSDH group than in the Blank group, as measured by WB and qPCR (Fig. 2D and E).

### HucMSCs successfully overexpressed keap1

**Fig. 3:**
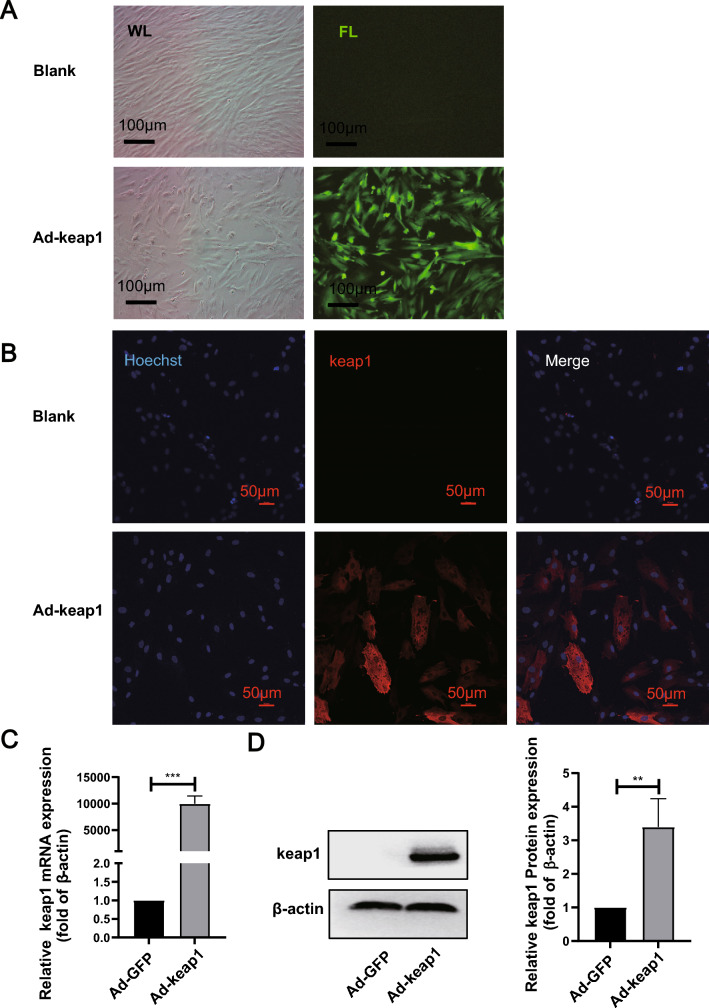
Successful overexpression of keap1. **A** Green fluorescence of cells was observed under inverted microscope. *WL* white light, *FL* fluorescence light. Scale bar: 100 μm. **B** Immunofluorescence detection of keap1 expression. Scale bar: 50 μm. **C** RT-qPCR detected keap1 mRNA level. p = 0.0079. **D** Quantification of keap1 protein level was measured by western blotting. p = 0.0003. (Colour figure online)

To explore the effect of keap1 on hucMSCs, we transfected hucMSCs using an adenovirus overexpressing the keap1 gene carrying GFP (Ad-keap1). 36 h after transfection, the cells were observed with bright green fluorescence under an inverted microscope (Fig. 3A). And the cellular immunofluorescence results showed that keap1 expression was significantly elevated in the Ad-keap1 group compared with the blank group (Blank) (Fig. 3B). As illustrated in Fig. 3C and D, the expression of both mRNA and protein of keap1 was significantly elevated after transfection of the virus compared with that of the null group (Ad-GFP group). The above results indicated that keap1 was successfully overexpressed in hucMSCs.

### Overexpression of keap1 caused a decrease in the proliferative capacity of hucMSCs

**Fig. 4:**
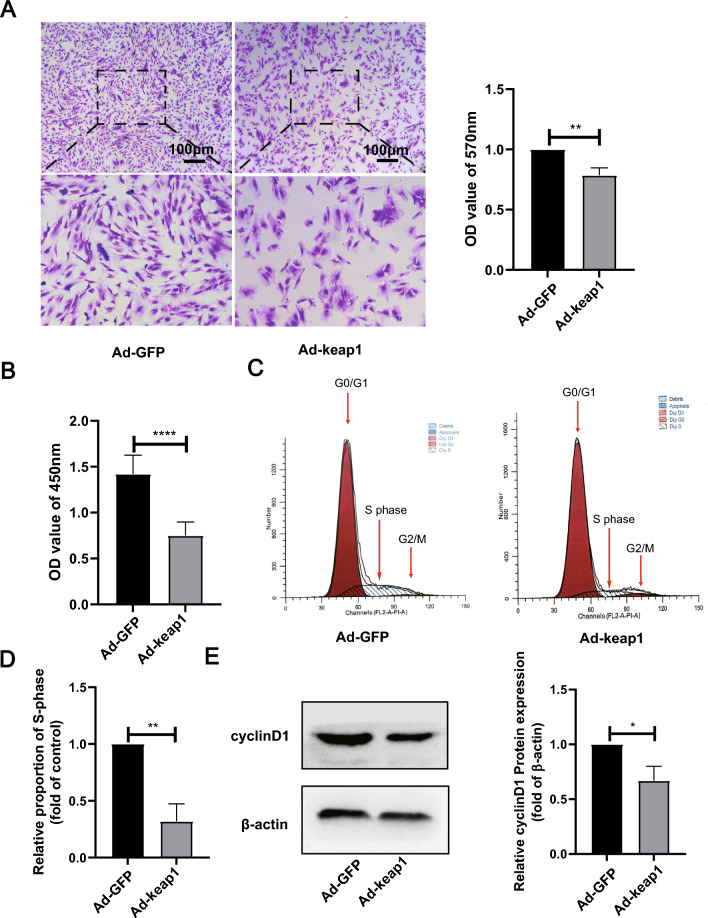
Effect of overexpression of keap1 on hucMSCs cell proliferation. **A** Crystalline violet staining as well as quantitative detection at 570 nm, scale bar: 100 μm. p = 0.0038. **B** CCK8 detected cell viability. n = 15. p < 0.0001. **C**, **D** PI staining was used to measured cell cycle and the proportion of S phase was quantified. p = 0.0016. **E** CyclinD1 protein level was detected by WB and quantified. p = 0.0121

As illustrated in Fig. 4A, the number of cells in the Ad-keap1 group was significantly reduced compared with that in the Ad-GFP group, assessed by crystal violet staining. CCK8 cell viability assay displayed that cell viability in the Ad-keap1 group was substantially suppressed compared with that in the Ad-GFP group (Fig. 4B). Meanwhile, As shown in Fig. 4C and D, the proportion of cells in S phase was significantly decreased in the Ad-keap1 group compared with the Ad-GFP group. Furthermore, the expression of cell cycle-related protein cyclinD1 was significantly decreased in the Ad-keap1 group compared with the Ad-GFP group (Fig. 4E).

### Overexpression of keap1 induced to oxidative stress in hucMSCs

**Fig. 5:**
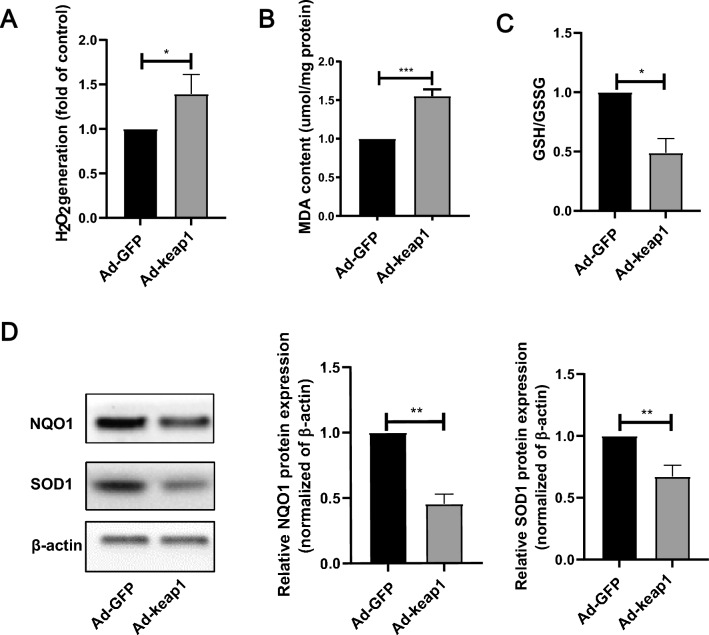
Overexpression of keap1 leads to oxidative stress in hucMSCs. **A** Amplex Red detected H_2_O_2_ production. p = 0.0358. **B** Lipid oxidation kit measured MDA level. p = 0.0004. **C** GSH and GSSG Assay Kit detected the ratio of reduced glutathione (GSH) and oxidized glutathione (GSSG). p = 0.0131. **D** WB detected the antioxidant protein SOD1 and NQO1 protein level. Statistical analysis is shown at right, p(NQO1) = 0.0018, p(SOD1) = 0.0033

Keap1 is important in regulating oxidative stress, so we further explored whether overexpression of keap1 leading to cellular injury was related to oxidative stress. As expected, keap1 overexpression increased H_2_O_2_ content (Fig. 5A) and lipid peroxidation product MDA accumulation (Fig. 5B), as well as a dramatic reduction in the ratio of reduced glutathione (GSH) to oxidized glutathione (GSSG) (Fig. 5C). As demonstrated in Fig. 5D, the protein levels of antioxidant enzyme NQO1 and SOD1 was significantly reduced in the Ad-keap1 group compared to the Ad-GFP group.

### Overexpression of keap1 inhibited ATP production

**Fig. 6:**
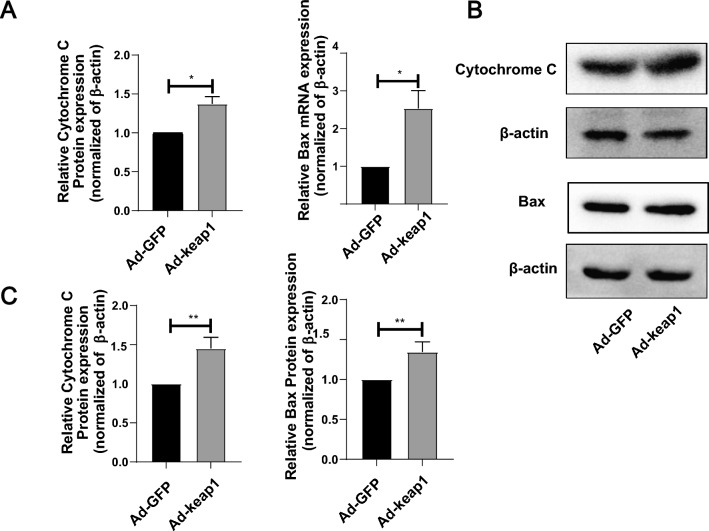
Effect of overexpression of keap1 on mitochondria of hucMSCs. **A** RT-qPCR detected the mRNA levels of cytochrome C, Bax. p(cytochrome C) = 0.0167, p(Bax) = 0.0315. **B**, **C** WB detected protein levels of cytochrome C, Bax. Statistical analysis is displayed below, p (cytochrome C) = 0.0056, p(Bax) = 0.0097

**Fig. 7:**
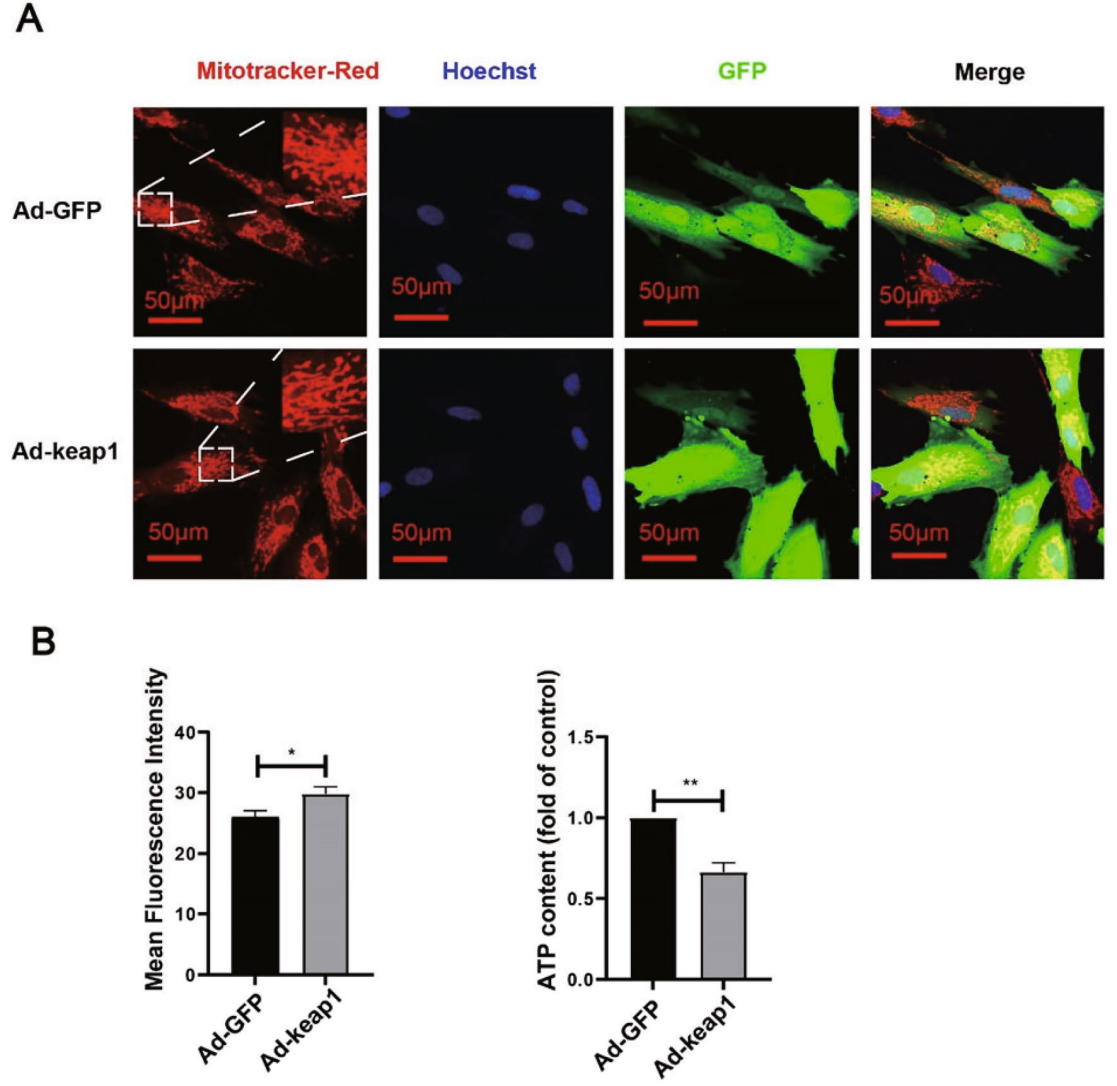
Effect of overexpression of keap1 on mitochondria of hucMSCs. **A** Mitotracker Red detected cellular mitochondrial morphology. Mitotracker Red stained mitochondria, Hoechst stained nuclei, and cells were infected with adenovirus carrying GFP. Image J was used to quantify the mean Mitotracker-Red Fluorescence intensity. p = 0.0198. Scale bar: 50 μm. **B** ATP Assay Kit detected ATP content. p = 0.1052. (Colour figure online)

H_2_O_2_ production is closely related to mitochondria, so we further investigated the effect of overexpression of keap1 on mitochondria. As demonstrated in Fig. 6A, B and C, keap1 overexpression upregulated the mRNA and protein levels of cytochrome C and Bax. Meanwhile, overexpression of keap1 increased the number of mitochondria but decreased ATP production (Fig. 7A and B).

### Overexpression of keap1 downregulates IKKβ expression and inhibits the NFκB pathway

**Fig. 8:**
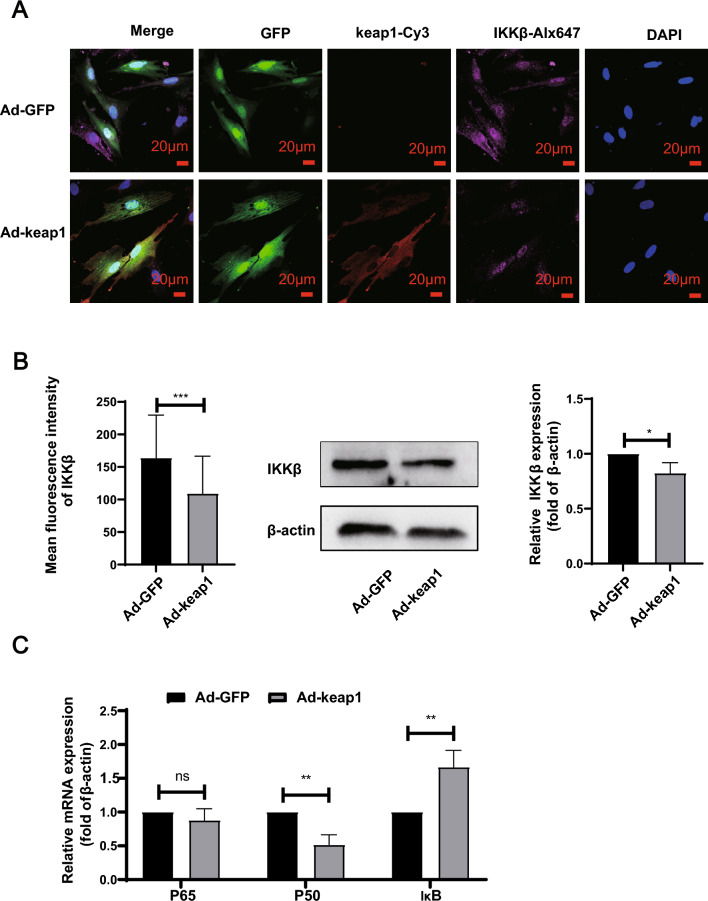
Overexpression of keap1 resulted in downregulation of IKKβ protein in hucMSCs. **A** Cellular immunofluorescence was used to detect the expression of keap1 and IKKβ protein levels, scale bar: 20 μm. p < 0.0001. **B** WB detected the expression level of IKKβ protein. p = 0.0324. **C** RT-qPCR measured the mRNA levels of P65, P50, IκB. P65: p = 0.2877, P50:p = 0.0046, IκB:p = 0.0092

In addition, we detected IKKβ, a protein that may interact with keap1. As shown in Fig. 8A and B, the cellular immunofluorescence and WB revealed that the overexpression of keap1 significantly diminished IKKβ expression. RT-qPCR illustrated that the NF-κB pathway was inhibited in the Ad-keap1 group compared to the Ad-GFP group, as evidenced by a decrease in P50 and an increase in IκB. (Fig. 8C).

## Discuss

Cells experience oxidative stress due to an imbalance in the ROS formation and scavenging system, which is directly related to cell death [9]. Ischemia, hypoxia and poor nutrition at the damaged site result in low implantation and survival rates of hucMSCs after transplantation into the body, which leads to poor efficacy [17, 18]. Therefore, the development of strategies to enhance hucMSCs implantation and survival rates is key to current stem cell therapy research. Currently, the strategies to enhance stem cell therapy are: pretreatment, gene modification [19]. Among them, gene modification targets genes by precise targeting for the purpose of enhancing efficacy [20]. With 624 amino acids, keap1 has a lot of cysteine residues. It plays a significant role in a number of biological processes, including cell proliferation [11], death, and especially oxidative stress. Keap1 functions as a biosensor for oxidative stress due to the presence of highly reactive cysteine residues. When cells are under stress, the reactive cysteine residues of keap1 are oxidized and its own activity is inhibited, which activates a series of cytoprotective genes [10]. Bibo Ke et al. [12]suggested that in the hepatic ischemia/reperfusion injury model, the inhibition of keap1 expression could promote antioxidant protein expression to resist oxidative stress injury induced by ischemia-reperfusion. Therefore, keap1 may be a potential target for improving stem cell therapy.

In our study, we found that when a hypoxic low-glucose serum-free model was used to simulate the damaged microenvironment, hucMSCs showed decreased proliferative capacity and increased ROS production, along with a significant rise in keap1. Subsequently, after overexpression of keap1 using adenovirus, it was discovered that overexpression of keap1 induced oxidative stress in hucMSCs, led to cell cycle arrest, and decreased proliferative capacity accompanied by IKKβ downregulation.

Cell cycle and cell proliferation are closely linked, and growth signaling regulates cell proliferation by targeting cell cycle-related proteins [21]. According to a research, keap1 knockdown prevents the production of cell cycle proteins and halts cell cycle progression in proliferating cells [22]. Contrarily, in our research, overexpression of keap1 suppressed cyclinD1 expression, resulting in hucMSCs blocking in the G1 phase and failing to enter the S phase (the period of DNA synthesis). Cell cycle arrest results from the suppression of the production of cyclinD1, a crucial regulator of the G1-S cell cycle transition [23]. Moreover, cyclinD1 is a target protein of the NF-κB pathway, and when the NF-κB pathway is inhibited, cyclinD1 is down-regulated causing the cells to block in G1 phase [24]. We therefore suggest that the reason for this discrepancy may be that overexpression of keap1 in hucMSCs down-regulates IKKβ and inhibits the activation of the NF-κB pathway, thereby inhibiting cell cycle progression.

The organism has a very fine regulation of ROS, so the organism has its own redox threshold, within which is a favorable stimulus for the organism to promote cell growth and development, while once the threshold is exceeded it leads to oxidative stress. Degradation of keap1 has been reported to mediate anti-oxidative stress [25, 26]. Hydrogen peroxide (H_2_O_2_) is one of the most studied ROS isoforms [27]. Keap1 induces hucMSCs to produce large amounts of H_2_O_2,_ and its aberrant accumulation may lead to oxidative stress. Oxidative stress leads to lipid peroxidation, resulting in MDA accumulation. Glutathione metabolism, an important cellular antioxidant defense system, may act synergistically with nicotinamide adenine dinucleotide phosphate (NADPH) to regulate and maintain the cellular redox state, degrade H_2_O_2_ and resist lipid peroxidation [28]. However, overexpression of keap1 decreased the ratio of reduced to oxidized glutathione(GSH/GSSG), reduced expression of antioxidant enzymes NQO1 and SOD1, thereby weakening cellular antioxidant effects and inablity to resist cellular oxidative stress damage.

Mitochondria produce the majority of H_2_O_2_, and its structural and functional abnormalities is one of the causes causing oxidative stress [29]. When apoptotic factors BAX and BAK are activated, it increases mitochondrial permeability and causes cytochrome C release, which leads to apoptosis [30]. Deletion of BAX and BAK, in contrast, increases MSC survival [31]. In our investigation, keap1 overexpression dramatically raised the BAX and cytochrome C expression. The powerhouses of the cell, the mitochondria, use oxygen to create ATP, which powers all of the important functions of the cell [32]. Tawfeeq Shekh-Ahmad et al. reported that acute inhibition of keap1 stimulated mitochondrial bioenergetics, increased glutathione and ATP to exert neuroprotection [33]. Interestingly, overexpression of keap1 increased the number of mitochondria but inhibited ATP production in our study. Why the change in the number of mitochondria does not match the change in ATP production, we suppose that it may be due to the increase in impaired mitochondria, which results in the cell not only failing to efficiently utilize oxygen to produce ATP, but also producing large amounts of H_2_O_2_.

The NF-κB pathway is critical in regulating intracellular ROS. IKKβ is an IκB kinase that activates the NF-κB pathway to activate anti-oxidative stress [15]. IKKβ serves as a substrate for the keap1-E3 ubiquitin ligase, which associates keap1 with the NF-κB pathway [34]. Keap1 induces IKKβ degradation and inhibition of the NF-κB pathway, which was related to cell proliferation [14]. It has been shown that activation of the NF-κB pathway inhibits apoptosis in MSC cells [6]. Interestingly, in our study, overexpression of keap1 was accompanied by a decrease in IKKβ and inhibition of the NF-κB pathway. However, whether IKKβ is a major factor in oxidative stress injury caused by overexpression of keap1 deserves further in-depth study.

## Conclusion

Keap1 occupies a very important position in oxidative stress injury of hucMSCs and is expected to be a potential target for genetic engineering of hucMSCs in the future, which brings new hope for the future development of new commercialized stem cell preparations, thus improving the efficacy of refractory childhood diseases.

## Data Availability

The authors confirm that the dataset supporting the conclusions of this article are included within the article.
